# Supplementary material for: Solvophobicity-directed assembly of microporous molecular crystals
Source: Commun Chem. 2021 Aug 20;4:122. doi: 10.1038/s42004-021-00561-8 (PMC9814291; doi:10.1038/s42004-021-00561-8)
Supplement: Supplementary file 1 — Supplementary Information [file 42004_2021_561_MOESM1_ESM.pdf]

Supplementary Information

Solvophobicity-directed assembly of microporous molecular crystals

Hiroshi Yamagishi*, Monika Tsunoda, Kohei Iwai, Kowit Hengphasatporn, Yasuteru Shigeta, Hiroyasu Sato, and Yohei Yamamoto

Department of Materials Science, Faculty of Pure and Applied Sciences, and Tsukuba Research Center for Energy Materials Science (TREMS), University of Tsukuba, 1-1-1 Tennodai, Tsukuba, Ibaraki 305-8573, Japan.

Center for Computational Sciences, University of Tsukuba, 1-1-1 Tennodai, Tsukuba, Ibaraki 305-8577, Japan.

Rigaku Corporation, 12-9-3 Matsubara, Akishima, Tokyo 196-8666, Japan.

*Correspondence and requests for materials should be addressed to Hiroshi Yamagishi (yamagishi.hiroshi.ff@u.tsukuba.ac.jp).

Table of Contents

1. Supplementary methods S3
2. Supplementary figures and tables S7
3. Supporting references S43

1. Supplementary methods

Synthesis of *m*-Py$_6$Mes and Ph$_6$Mes

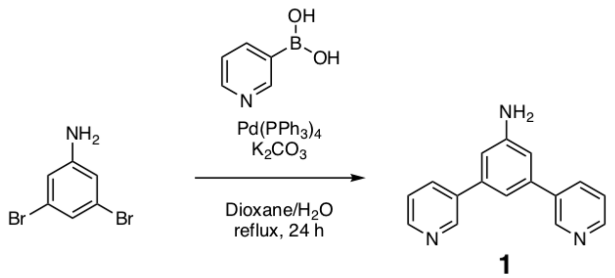

Reaction procedure for 1: To a Schlenk flask purged with dibromoaniline (850 mg), pyridine boronic acid (1.1 g), and K_2CO_3 (2.6 g) in a mixture of degassed dioxane (30 mL) and H_2O (7.5 mL) under N_2 gas was added $Pd(PPh_3)_4$ (270 mg). The resulting solution was refluxed for 24 h. After cooling to ambient temperature, the resulting suspension was collected and dried under reduced pressure. The resultant crude was dissolved in $CHCl_3$ and extracted with aqueous solution of HCl. To the aqueous phase was added aqueous solution of NaOH dropwise until the extract was neutralized. Resultant precipitate was collected through cellulose filter and was dried under reduced pressure to yield **1** as brown solid (900 mg). The product was utilized for the next reaction without further purification. ^{1}H NMR (400 MHz, $CDCl_3$): δ (ppm) 4.05 (s, 2H, NH_2), 6.86 (s, 2H, Ar), 7.06 (s, 1H, Ar), 7.31 (dd, J = 4.4, 8.0 Hz, 2H, Ar), 7.83 (d, J = 8.0 Hz, 2H, Ar), 8.56 (d, J = 4.4 Hz, 2H, Ar), 8.80 (s, 2H, Ar).

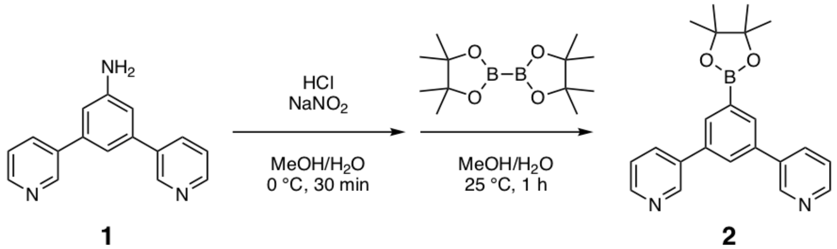

Reaction procedure for 2: To a round flask purged with **1** (250 mg) and HCl (262 mg) in a mixture of MeOH (2.0 mL) and H_2O (1.8 mL) was added aqueous solution of $NaNO_2$ (0.5 mL, 2.2

M) dropwise at 0 °C. The mixture was stirred for 30 min at 0 °C under atmosphere. After being heated up to 25 °C, MeOH solution of bis(pinacolato)diboron (5.0 mL, 0.6 M) was added to the solution. The mixture was stirred at 25 °C for 60 min. The resultant solution was washed with CH_2Cl_2. The aqueous layer was neutralized with aqueous solution of $NaHCO_3$, and the precipitate was collected through cellulose filter. The residue was purified on silica-gel column (CH_2Cl_2/MeOH = 95/5), and further purified with preparative GPC (eluent: $CHCl_3$) to yield **2** as yellow solid (50 mg, 14%). ^{1}H NMR (400 MHz, $CDCl_3$): δ (ppm) 1.36 (s, 12H, CH_3), 7.37 (dd, J = 4.4, 8.0 Hz, 2H, Ar), 7.84 (t, J = 2.0 Hz, 1H, Ar), 7.95 (dt, J = 2.0, 8.0 Hz, 2H, Ar), 8.04 (d, J = 2.0 Hz, 2H, Ar), 8.60 (d, J = 3.6 Hz, 2H, Ar), 8.91 (s, 2H, Ar).

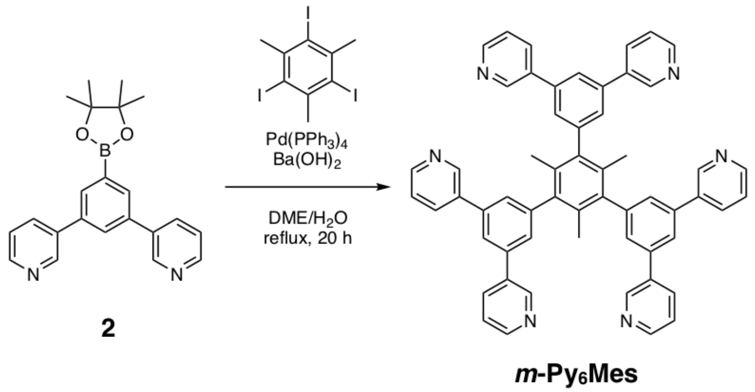

Reaction procedure for *m*-Py$_6$Mes: To a Schlenk flask purged with **2** (32 mg), triiodomesitylene (9.8 mg) and $Ba(OH)_2{\bullet}8H_2O$ (28 mg) in a mixture of degassed dimethoxyethane (1.8 mL) and H_2O (0.45 mL) under N_2 was added $Pd(PPh_3)_4$ (6.0 mg). The resulting solution was refluxed for 20 h. The resulting suspension was collected and extracted with CH_2Cl_2 for two times. The combined organic layer was dried under reduced pressure and was purified on silica-gel column (CH_2Cl_2/MeOH = 95/5), and further purified with preparative GPC (eluent: $CHCl_3$) to yield ***m*-Py$_6$Mes** as yellow solid (10.8 mg, 69%). ^{1}H NMR (400 MHz, $CDCl_3$): δ (ppm) 1.94 (s, 9H, CH_3), 7.39 (dd, J = 3.2, 5.2 Hz, 6H, Ar), 7.55 (d, J = 1.6 Hz, 6H, Ar), 7.78 (t, J = 1.6 Hz, 3H, Ar), 7.97 (dt, J = 2.0, 8.0 Hz, 6H, Ar), 8.62 (d, J = 2.0 Hz, 6H, Ar), 8.93 (d, J = 2.0 Hz, 6H, Ar), ^{13}C NMR (150 MHz, $CDCl_3$): δ (ppm) 20.0, 123.7, 124.4, 127.8, 133.6, 134.5, 136.0, 139.2, 139.4,

143.5, 148.3, 148.9. HRMS (ESI-FTMS) *m*/*z*: calcd. for $C_{57}H_{42}N_6$ $[M + H]^+$: 811.3544; found: 811.3528.

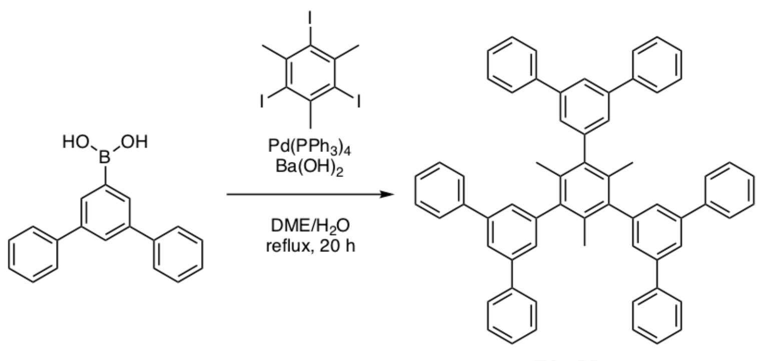

Reaction procedure for Ph$_6$Mes: To a Schlenk flask purged with N_2 filled with dimethoxyethane (9.5 mL) and H_2O (2.2 mL) was added $Pd(PPh_3)_4$ (46 mg), 5'-*m*-terphenylboronic acid (300 mg), triiodomesitylene (97 mg) and $Ba(OH)_2$•$8H_2O$ (307 mg) and was refluxed for 20 h. The resultant suspension was collected and extracted with CH_2Cl_2 for two times. The combined organic layer was dried under reduced pressure and was purified on a silica-gel column ($CHCl_3$/Hexane = 2/8) to yield **Ph$_6$Mes** as white solid (130 mg, 83%). ^{1}H NMR (400 MHz, $CDCl_3$): δ (ppm) 1.99 (s, 9H, CH_3), 7.38 (t, J = 7.6 Hz, 6H, Ar), 7.48 (t, J = 7.6 Hz, 12H, Ar), 7.55 (d, J = 1.2 Hz, 6H, Ar), 7.72 (d, J = 7.6 Hz, 12H, Ar), 7.84 (t, J = 1.6 Hz, 3H, Ar), ^{13}C NMR (100 MHz, $CDCl_3$): δ (ppm) 19.9, 124.2, 127.1, 127.2, 127.4, 128.8, 133.5, 139.9, 141.0, 141.9, 142.9; analysis (calcd., found for $C_{63}H_{48}$): C (93.99, 94.10), H (6.01, 5.88).

2. Supplementary figures and tables

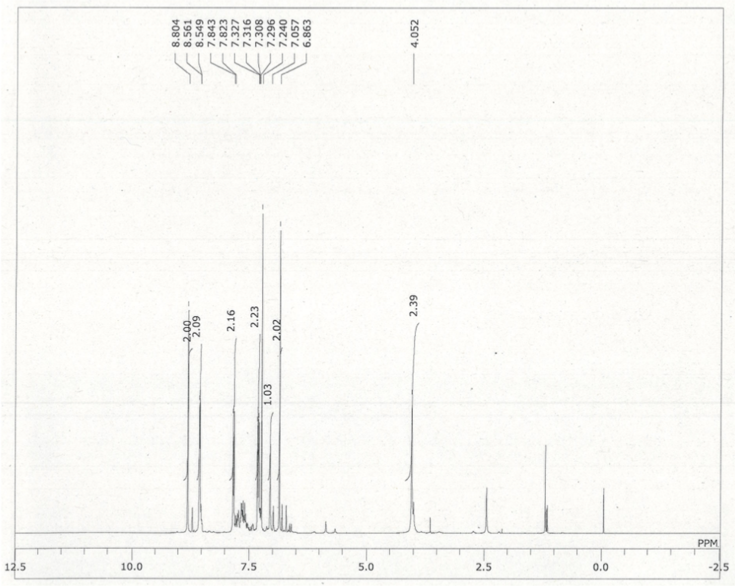

Supplementary Figure 1. ^{1}H NMR spectrum of **1** ($CDCl_3$, 400 MHz).

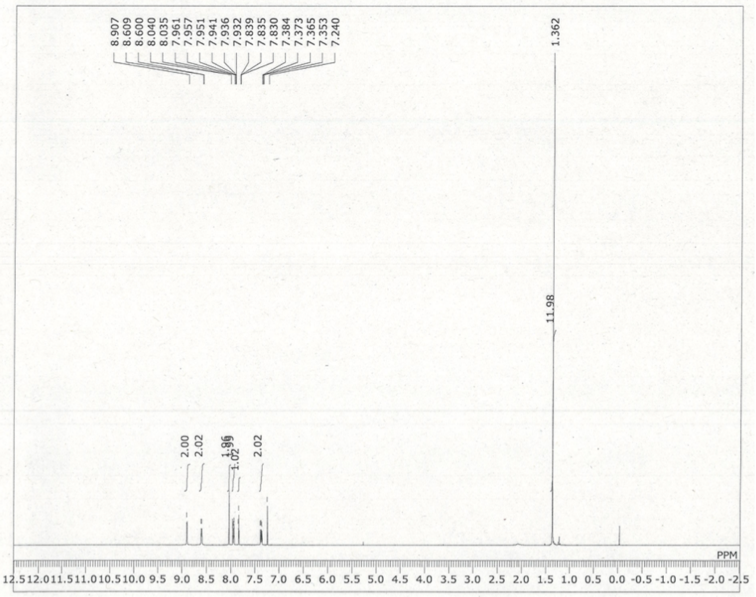

Supplementary Figure 2. ^{1}H NMR spectrum of **2** ($CDCl_3$, 400 MHz).

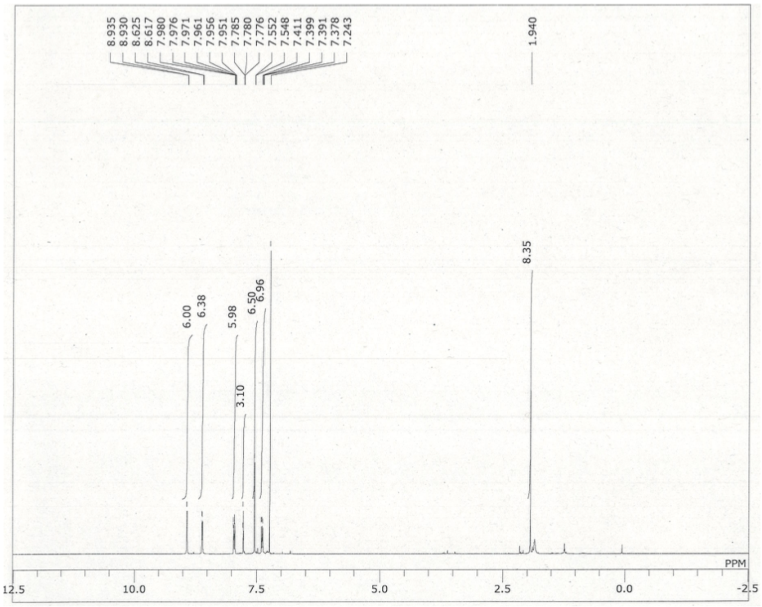

Supplementary Figure 3. ^{1}H NMR spectrum of ***m*-Py$_6$Mes** ($CDCl_3$, 400 MHz).

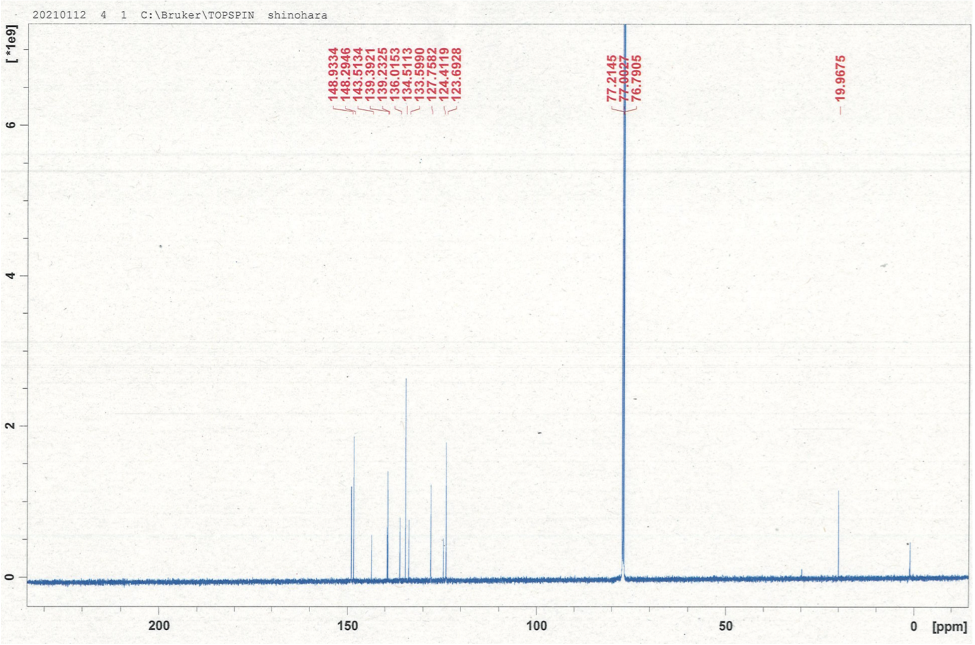

Supplementary Figure 4. ^{13}C NMR spectrum of ***m*-Py$_6$Mes** ($CDCl_3$, 150 MHz).

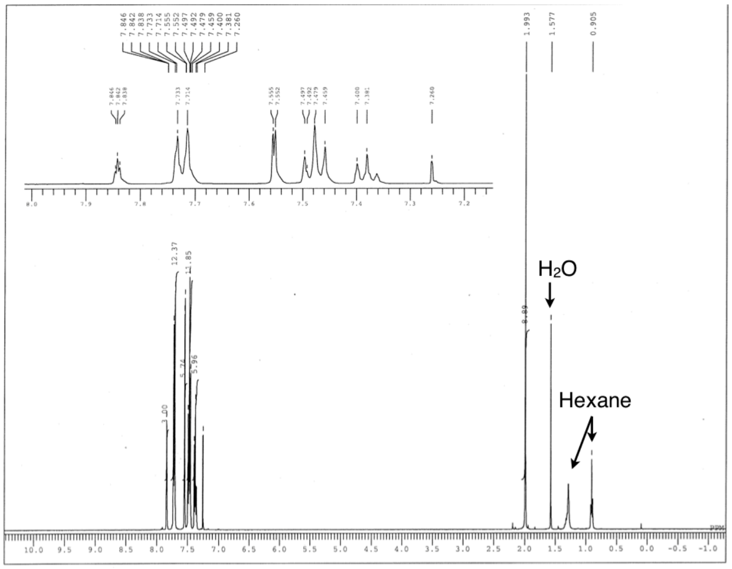

Supplementary Figure 5. ^{1}H NMR spectrum of **Ph$_6$Mes** ($CDCl_3$, 400 MHz).

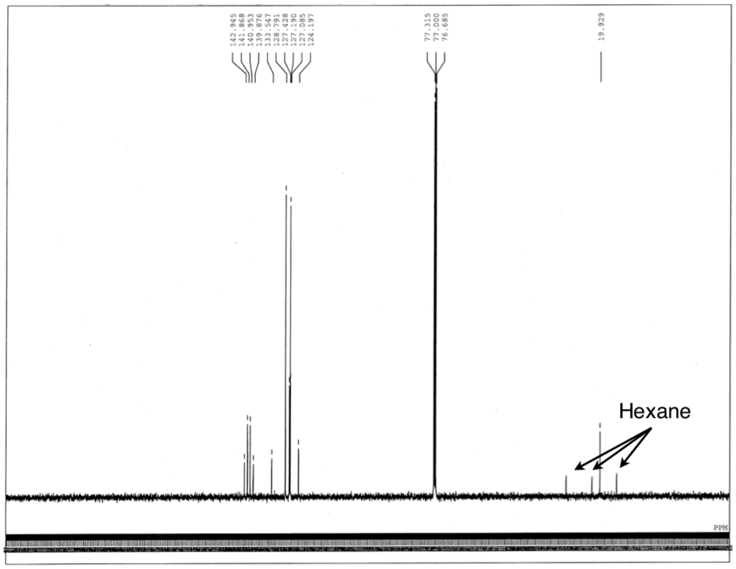

Supplementary Figure 6. ^{13}C NMR spectrum of **Ph$_6$Mes** ($CDCl_3$, 100 MHz).

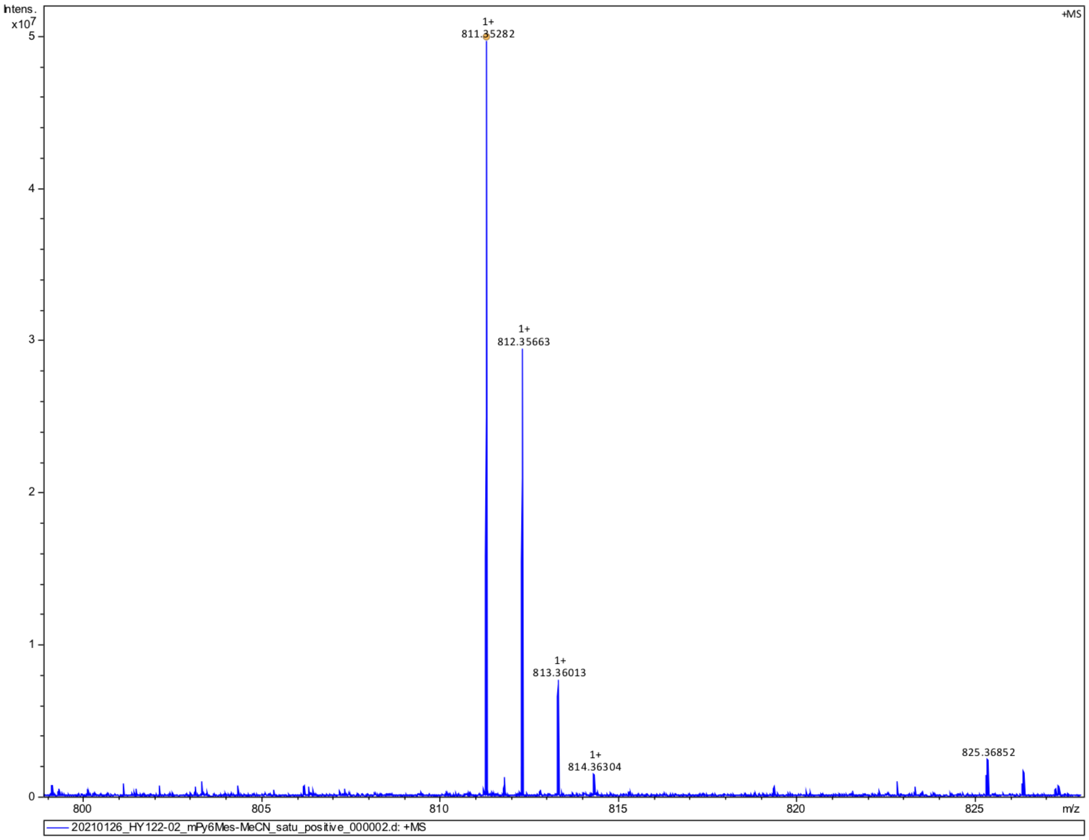

Supplementary Figure 7. High resolution ESI-FTMS of ***m*-Py$_6$Mes**.

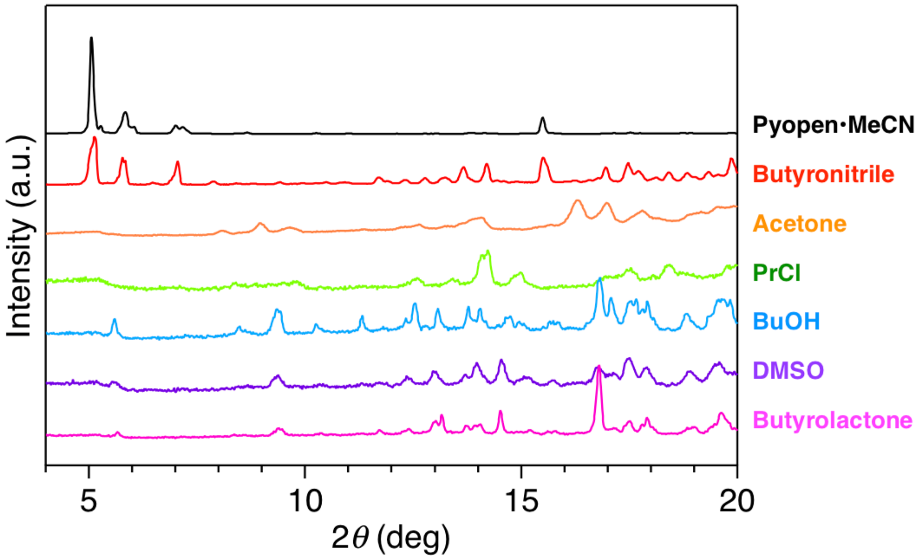

Supplementary Figure 8. Powder X-ray diffraction profiles of **Py$_6$Mes** crystallized in MeCN (black), butyronitrile (red), acetone (orange), 1-chloropropane (green), 1-butanol (blue), dimethylsulfoxide (purple), and butyrolactone (pink).

Supplementary Table 1. Crystal structure information of **Py^{open}•EtOAc.**

Crystal data	
Chemical formula	$C_{57}H_{42}N_6$
M_r	811.00
Crystal system, space group	Monoclinic, $P2_1/c$
Temperature (K)	273
a, *b*, *c* (Å)	17.206 (4), 10.531 (2), 30.275 (8)
β (°)	99.893 (4)
V (Å^3)	5404 (2)
Z	4
Radiation type	Mo $K\alpha$
μ (mm^{-1})	0.06
Crystal size (mm)	0.30 × 0.24 × 0.23
Data collection	
Diffractometer	Rigaku Saturn70
Absorption correction	Multi-scan<br>REQAB (Rigaku, 1998)
T_{min}, T_{max}	0.519, 0.986
No. of measured, independent and observed [$F^2 > 2.0\sigma(F^2)$] reflections	41071, 12237, 5901
R_{int}	0.112
$(\sin \theta/\lambda)_{max}$ (Å^{-1})	0.649
Refinement	
$R[F^2 > 2\sigma(F^2)]$, $wR(F^2)$, S	0.090, 0.301, 1.02
No. of reflections	12237
No. of parameters	571
H-atom treatment	H-atom parameters constrained
$\Delta\rho_{max}$, $\Delta\rho_{min}$ (e Å^{-3})	0.25, −0.28

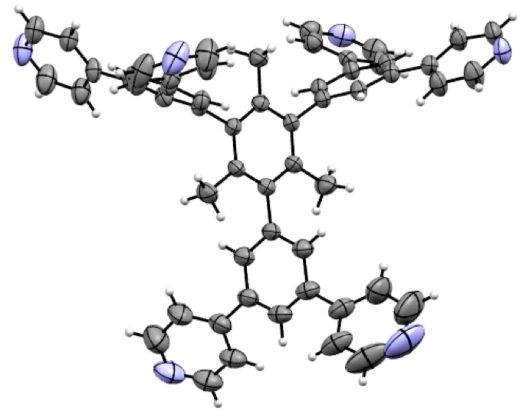

Supplementary Figure 9. An ORTEP diagram of **Py^{open}**•EtOAc with a probability level of 50 %.

Supplementary Table 2. Crystal structure information of **Py^{VDW}•CH_2Cl_2.**

Crystal data	
Chemical formula	$C_{57}H_{42}N_6$•2(CH_2Cl_2)
M_r	980.86
Crystal system, space group	Monoclinic, *C*2/*c*
Temperature (K)	93
a, *b*, *c* (Å)	16.946 (5), 12.865 (4), 22.452 (8)
β (°)	100.461 (4)
V (Å^3)	4813 (3)
Z	4
Radiation type	Mo *K*α
μ (mm^{-1})	0.29
Crystal size (mm)	0.40 × 0.35 × 0.15
Data collection	
Diffractometer	Rigaku Saturn70
Absorption correction	Multi-scan<br>REQAB (Rigaku, 1998)
T_{min}, T_{max}	0.849, 0.957
No. of measured, independent and observed [$F^2 > 2.0\sigma(F^2)$] reflections	18352, 5432, 5175
R_{int}	0.033
$(\sin \theta/\lambda)_{max}$ (Å^{-1})	0.648
Refinement	
$R[F^2 > 2\sigma(F^2)]$, $wR(F^2)$, S	0.053, 0.131, 1.10
No. of reflections	5432
No. of parameters	316
H-atom treatment	H-atom parameters constrained
$\Delta\rho_{max}$, $\Delta\rho_{min}$ (e Å^{-3})	0.33, −0.46

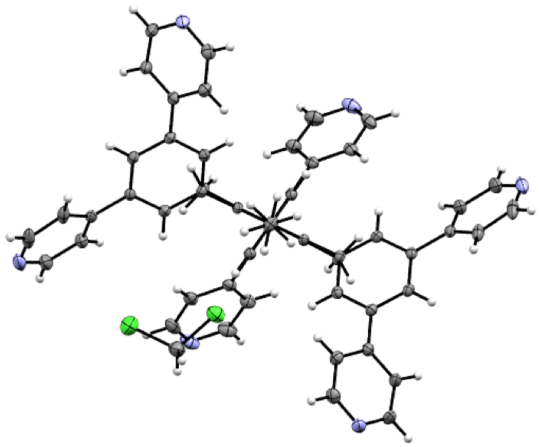

Supplementary Figure 10. An ORTEP diagram of $\mathbf{Py^{VDW} \bullet CH_2Cl_2}$ with a probability level of 50 %.

Supplementary Table 3. Crystal structure information of **Py^{VDW}•C_7H_8.**

Crystal data	
Chemical formula	$C_{57}H_{42}N_6$•C_7H_8
M_r	903.14
Crystal system, space group	Triclinic, $P\bar{1}$
Temperature (K)	273
a, *b*, *c* (Å)	16.633 (5), 18.110 (6), 18.159 (6)
α, β, γ (°)	91.3845 (10), 106.522 (5), 110.295 (4)
V (Å^3)	4872 (3)
Z	4
Radiation type	Mo $K\alpha$
μ (mm^{-1})	0.07
Crystal size (mm)	0.30 × 0.30 × 0.03
Data collection	
Diffractometer	Rigaku Saturn70
Absorption correction	Multi-scan<br>REQAB (Rigaku, 1998)
T_{min}, T_{max}	0.841, 0.998
No. of measured, independent and observed [$F^2 > 2.0\sigma(F^2)$] reflections	41360, 21433, 16316
R_{int}	0.064
$(\sin \theta/\lambda)_{max}$ (Å^{-1})	0.649
Refinement	
$R[F^2 > 2\sigma(F^2)]$, $wR(F^2)$, S	0.121, 0.296, 1.14
No. of reflections	21433
No. of parameters	1269
H-atom treatment	H-atom parameters constrained
$\Delta\rho_{max}$, $\Delta\rho_{min}$ (e Å^{-3})	0.70, −0.44

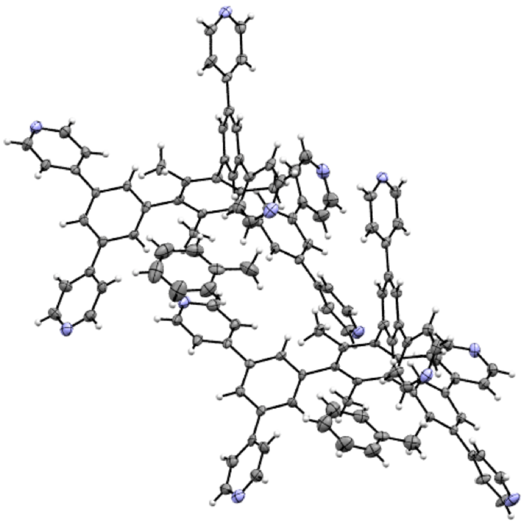

Supplementary Figure 11. An ORTEP diagram of $\mathbf{Py^{VDW}{\bullet}C_7H_8}$ with a probability level of 50 %.

Supplementary Table 4. Crystal structure information of ***m*-Py^{VDW}•MeCN.**

Crystal data	
Chemical formula	$2(C_{57}H_{42}N_6)$
M_r	1621.93
Crystal system, space group	Triclinic, $P\overline{1}$
Temperature (K)	93
a, b, c (Å)	11.701, 19.241, 21.820
α, β, γ (°)	107.22, 104.21, 104.14
V (Å^3)	4273.9
Z	2
Radiation type	Mo $K\alpha$
μ (mm^{-1})	0.08
Crystal size (mm)	150 × 120 × 100
Data collection	
Diffractometer	Bruker *SMART APEX2* area detector
Absorption correction	Multi-scan
T_{min}, T_{max}	0.660, 0.746
No. of measured, independent and observed [$I > 2\sigma(I)$] reflections	24683, 18637, 14834
R_{int}	0.014
$(\sin \theta/\lambda)_{max}$ (Å^{-1})	0.649
Refinement	
$R[F^2 > 2\sigma(F^2)]$, $wR(F^2)$, S	0.045, 0.122, 1.03
No. of reflections	18637
No. of parameters	1141
H-atom treatment	H-atom parameters constrained
$\Delta\rho_{max}$, $\Delta\rho_{min}$ (e Å^{-3})	0.35, −0.26

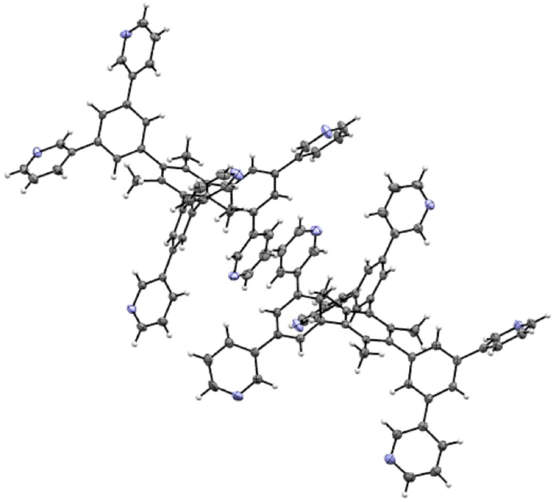

Supplementary Figure 12. An ORTEP diagram of ***m*-Py$^{\mathbf{VDW}}$•MeCN** with a probability level of 50 %.

Supplementary Table 5. Crystal structure information of ***m*-Py^{VDW}•EtOAc.**

Crystal data	
Chemical formula	$2(C_{57}H_{42}N_6)$
M_r	1621.93
Crystal system, space group	Triclinic, $P\overline{1}$
Temperature (K)	93
a, *b*, *c* (Å)	11.6490 (13), 19.114 (2), 21.707 (2)
α, β, γ (°)	107.133 (2), 104.165 (2), 104.290 (2)
V (Å^3)	4204.7 (8)
Z	2
Radiation type	Mo $K\alpha$
μ (mm^{-1})	0.08
Crystal size (mm)	90 × 60 × 10
Data collection	
Diffractometer	Bruker *SMART APEX2* area detector
Absorption correction	Multi-scan<br>*SADABS2016*/2 (Bruker,2016/2) was used for absorption correction. wR2(int) was 0.0867 before and 0.0415 after correction. The Ratio of minimum to maximum transmission is 0.8898. The λ/2 correction factor is Not present.
T_{min}, T_{max}	0.663, 0.745
No. of measured, independent and observed [$I > 2\sigma(I)$] reflections	22155, 16095, 7307
R_{int}	0.046
$(\sin \theta/\lambda)_{max}$ (Å^{-1})	0.616
Refinement	
$R[F^2 > 2\sigma(F^2)]$, $wR(F^2)$, S	0.072, 0.203, 0.97
No. of reflections	16095
No. of parameters	1141
H-atom treatment	H-atom parameters constrained
$\Delta\rho_{max}$, $\Delta\rho_{min}$ (e Å^{-3})	0.46, −0.26

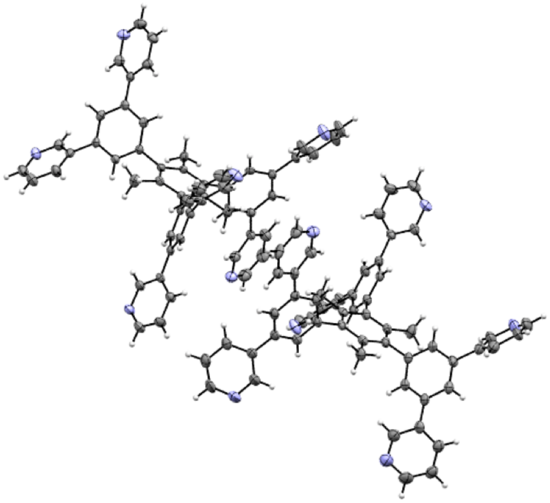

Supplementary Figure 13. An ORTEP diagram of ***m*-Py$^{\mathbf{VDW}}$•EtOAc** with a probability level of 50 %.

Supplementary Table 6. Crystal structure information of ***m*-Py^{VDW}•*i*PA.**

Crystal data	
Chemical formula	$C_{57}H_{42}N_6 \cdot 2(C_3H_8O)$
M_r	931.15
Crystal system, space group	Triclinic, $P\overline{1}$
Temperature (K)	93
a, *b*, *c* (Å)	9.5699 (3), 15.0232 (5), 19.3749 (7)
α, β, γ (°)	68.846 (3), 85.113 (3), 79.339 (3)
V (Å^3)	2552.48 (16)
Z	2
Radiation type	Cu *K*α
μ (mm^{-1})	0.58
Crystal size (mm)	300.0 × 100.0 × 10.0
Data collection	
Diffractometer	Rigaku XtaLAB P200
Absorption correction	Multi-scan<br>*CrysAlis PRO* 1.171.40.72a (Rigaku Oxford Diffraction, 2020) Empirical absorption correction using spherical harmonics, implemented in SCALE3 ABSPACK scaling algorithm.
T_{min}, T_{max}	0.599, 1.000
No. of measured, independent and observed [$I > 2\sigma(I)$] reflections	28129, 9733, 8498
R_{int}	0.068
$(\sin \theta/\lambda)_{max}$ (Å^{-1})	0.619
Refinement	
$R[F^2 > 2\sigma(F^2)]$, $wR(F^2)$, S	0.082, 0.235, 1.05
No. of reflections	9733
No. of parameters	823
H-atom treatment	H atoms treated by a mixture of independent and constrained refinement
$\Delta\rho_{max}$, $\Delta\rho_{min}$ (e Å^{-3})	0.90, −0.69

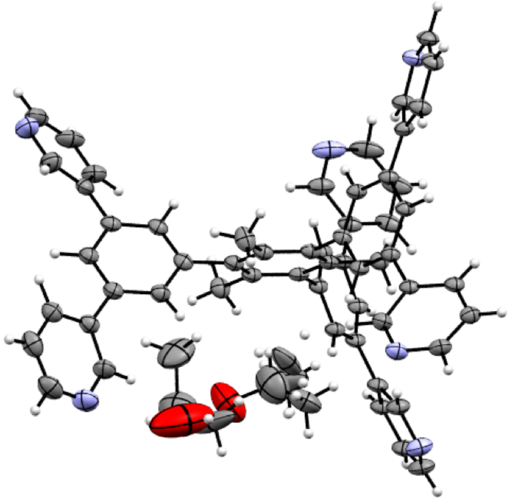

Supplementary Figure 14. An ORTEP diagram of ***m*-Py$^{\mathbf{VDW}}$•*i*PA** with a probability level of 50 %.

Supplementary Table 7. Crystal structure information of ***m*-Py^{VDW}•$CHCl_3$.**

Crystal data	
Chemical formula	$2(CHCl_3)\cdot C_{57}H_{42}N_6$
M_r	1049.70
Crystal system, space group	Monoclinic, $P2_1/n$
Temperature (K)	93
a, *b*, *c* (Å)	11.5262 (8), 25.3574 (17), 17.7834 (12)
β (°)	102.282 (1)
V (Å^3)	5078.7 (6)
Z	4
Radiation type	Mo *K*α
μ (mm^{-1})	0.39
Crystal size (mm)	80 × 20 × 10
Data collection	
Diffractometer	Bruker *SMART APEX2* area detector
Absorption correction	Multi-scan<br>*SADABS2016*/2 (Bruker,2016/2) was used for absorption correction. wR2(int) was 0.0509 before and 0.0309 after correction. The Ratio of minimum to maximum transmission is 0.9184. The λ/2 correction factor is Not present.
T_{min}, T_{max}	0.684, 0.745
No. of measured, independent and observed [$I > 2\sigma(I)$] reflections	25397, 9395, 6793
R_{int}	0.038
$(\sin \theta/\lambda)_{max}$ (Å^{-1})	0.605
Refinement	
$R[F^2 > 2\sigma(F^2)]$, $wR(F^2)$, S	0.045, 0.116, 1.01
No. of reflections	9395
No. of parameters	671
H-atom treatment	H-atom parameters constrained
$\Delta\rho_{max}$, $\Delta\rho_{min}$ (e Å^{-3})	0.56, −0.53

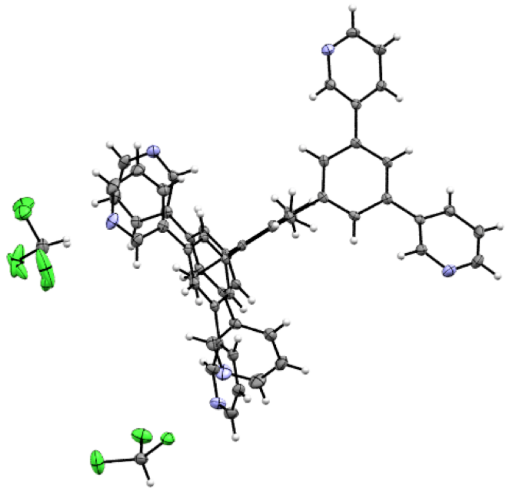

Supplementary Figure 15. An ORTEP diagram of ***m*-Py$^{\mathbf{VDW}}$•CHCl$_3$** with a probability level of 50 %.

Supplementary Table 8. Crystal structure information of $\mathbf{Ph^{VDW}}$**•MeCN.**

Crystal data	
Chemical formula	$C_{63}H_{48}$
M_r	805.01
Crystal system, space group	Triclinic, $P\overline{1}$
Temperature (K)	100
a, *b*, *c* (Å)	10.1054 (1), 11.3531 (2), 40.4637 (4)
α, β, γ (°)	93.305 (1), 90.584 (1), 110.178 (1)
V (Å^3)	4347.90 (10)
Z	4
Radiation type	Cu *K*α
μ (mm^{-1})	0.52
Crystal size (mm)	0.50 × 0.10 × 0.02
Data collection	
Diffractometer	Rigaku XtaLAB Synergy-Custom
Absorption correction	Multi-scan<br>*CrysAlis PRO* 1.171.41.110a (Rigaku Oxford Diffraction, 2021) Empirical absorption correction using spherical harmonics, implemented in SCALE3 ABSPACK scaling algorithm.
T_{min}, T_{max}	0.446, 1.000
No. of measured, independent and observed [$I > 2\sigma(I)$] reflections	55079, 17073, 14291
R_{int}	0.044
$(\sin \theta/\lambda)_{max}$ (Å^{-1})	0.626
Refinement	
$R[F^2 > 2\sigma(F^2)]$, $wR(F^2)$, S	0.047, 0.131, 1.05
No. of reflections	17073
No. of parameters	1141
H-atom treatment	H-atom parameters constrained
$\Delta\rho_{max}$, $\Delta\rho_{min}$ (e Å^{-3})	0.29, −0.34

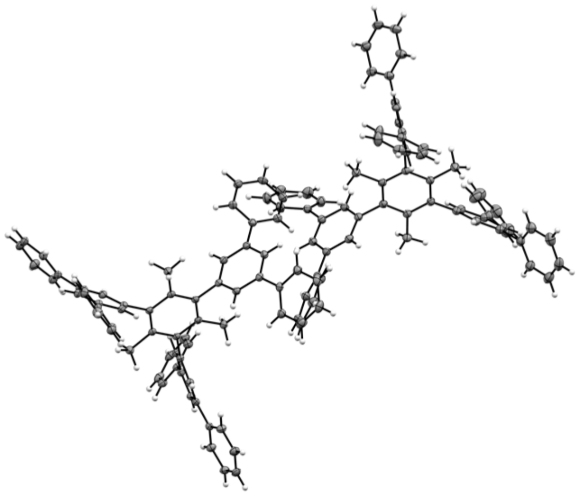

Supplementary Figure 16. An ORTEP diagram of **Ph^{VDW}•MeCN** with a probability level of 50 %.

Supplementary Table 9. Crystal structure information of **Ph^{VDW}•EtOAc.**

Crystal data	
Chemical formula	$C_{63}H_{48}\cdot C_4H_8O_2$
M_r	893.11
Crystal system, space group	Triclinic, $P\bar{1}$
Temperature (K)	100
a, *b*, *c* (Å)	11.2338 (2), 14.5022 (3), 17.3472 (3)
α, β, γ (°)	70.282 (2), 80.554 (2), 68.644 (2)
V (Å^3)	2474.99 (9)
Z	2
Radiation type	Cu *K*α
μ (mm^{-1})	0.54
Crystal size (mm)	0.45 × 0.28 × 0.22
Data collection	
Diffractometer	Rigaku XtaLAB Synergy-Custom
Absorption correction	Multi-scan<br>*CrysAlis PRO* 1.171.41.110a (Rigaku Oxford Diffraction, 2021) Empirical absorption correction using spherical harmonics, implemented in SCALE3 ABSPACK scaling algorithm.
T_{min}, T_{max}	0.649, 1.000
No. of measured, independent and observed [$I > 2\sigma(I)$] reflections	28171, 9732, 8656
R_{int}	0.065
$(\sin \theta/\lambda)_{max}$ (Å^{-1})	0.626
Refinement	
$R[F^2 > 2\sigma(F^2)]$, $wR(F^2)$, S	0.054, 0.160, 1.05
No. of reflections	9732
No. of parameters	627
H-atom treatment	H-atom parameters constrained
$\Delta\rho_{max}$, $\Delta\rho_{min}$ (e Å^{-3})	0.30, −0.42

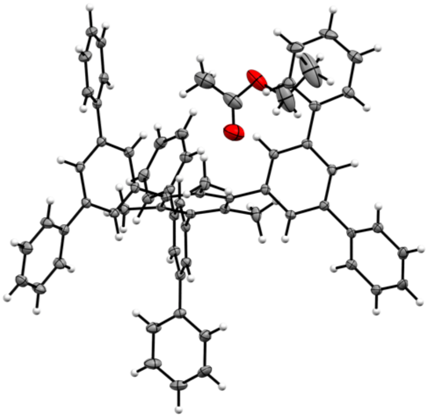

Supplementary Figure 17. An ORTEP diagram of **Ph^{VDW}• EtOAc** with a probability level of 50 %.

Supplementary Table 10. Crystal structure information of **Ph^{VDW}•THF.**

Crystal data	
Chemical formula	$2(C_4H_8O)\cdot C_{63}H_{48}$
M_r	949.22
Crystal system, space group	Monoclinic, *C*2/*c*
Temperature (K)	100
a, *b*, *c* (Å)	16.8279 (3), 12.9634 (2), 24.2308 (4)
β (°)	99.885 (2)
V (Å^3)	5207.40 (15)
Z	4
Radiation type	Cu *K*α
μ (mm^{-1})	0.54
Crystal size (mm)	0.37 × 0.31 × 0.13
Data collection	
Diffractometer	Rigaku XtaLAB Synergy-Custom
Absorption correction	Multi-scan<br>*CrysAlis PRO* 1.171.41.110a (Rigaku Oxford Diffraction, 2021) Empirical absorption correction using spherical harmonics, implemented in SCALE3 ABSPACK scaling algorithm.
T_{min}, T_{max}	0.568, 1.000
No. of measured, independent and observed [$I > 2\sigma(I)$] reflections	16182, 5093, 4737
R_{int}	0.021
$(\sin \theta/\lambda)_{max}$ (Å^{-1})	0.626
Refinement	
$R[F^2 > 2\sigma(F^2)]$, $wR(F^2)$, S	0.042, 0.111, 1.05
No. of reflections	5093
No. of parameters	345
H-atom treatment	H-atom parameters constrained
$\Delta\rho_{max}$, $\Delta\rho_{min}$ (e Å^{-3})	0.40, −0.33

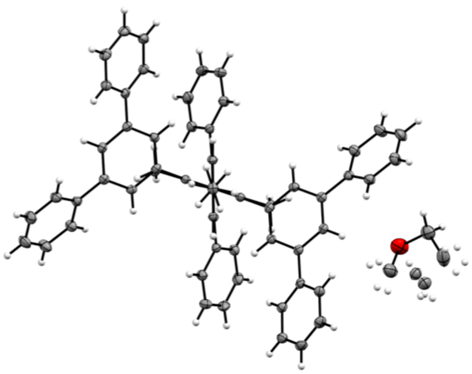

Supplementary Figure 18. An ORTEP diagram of $\mathbf{Ph^{VDW}}$**• THF** with a probability level of 50 %.

Supplementary Table 11. Crystal structure information of **Ph^{VDW}•$CHCl_3$.**

Crystal data	
Chemical formula	$2(CHCl_3)\cdot C_{63}H_{48}$
M_r	1043.75
Crystal system, space group	Monoclinic, *C*2/*c*
Temperature (K)	100
a, *b*, *c* (Å)	16.9997 (4), 13.1549 (3), 23.5266 (5)
β (°)	98.076 (2)
V (Å^3)	5209.1 (2)
Z	4
Radiation type	Cu *K*α
μ (mm^{-1})	3.33
Crystal size (mm)	0.67 × 0.56 × 0.28
Data collection	
Diffractometer	Rigaku XtaLAB Synergy-Custom
Absorption correction	Gaussian<br>*CrysAlis PRO* 1.171.41.110a (Rigaku Oxford Diffraction, 2021) Numerical absorption correction based on gaussian integration over a multifaceted crystal model Empirical absorption correction using spherical harmonics, implemented in SCALE3 ABSPACK scaling algorithm.
T_{min}, T_{max}	0.035, 1.000
No. of measured, independent and observed [$I > 2\sigma(I)$] reflections	14749, 5082, 4638
R_{int}	0.079
$(\sin \theta/\lambda)_{max}$ (Å^{-1})	0.626
Refinement	
$R[F^2 > 2\sigma(F^2)]$, $wR(F^2)$, S	0.090, 0.247, 1.03
No. of reflections	5082
No. of parameters	335
H-atom treatment	H-atom parameters constrained
$\Delta\rho_{max}$, $\Delta\rho_{min}$ (e Å^{-3})	0.89, −0.66

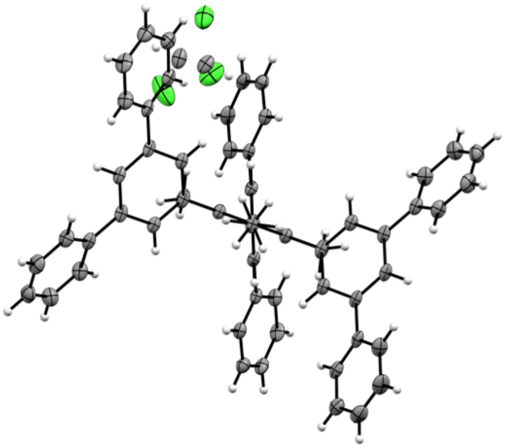

Supplementary Figure 19. An ORTEP diagram of $\mathbf{Ph^{VDW}}$**•** $\mathbf{CHCl_3}$ with a probability level of 50 %.

Supplementary Table 12. Crystal structure information of **Ph^{VDW}•C_7H_8.**

Crystal data	
Chemical formula	$C_{63}H_{48}·C_7H_8$
M_r	897.14
Crystal system, space group	Triclinic, $P\bar{1}$
Temperature (K)	100
a, *b*, *c* (Å)	11.2539 (3), 14.5508 (3), 17.3425 (4)
α, β, γ (°)	70.210 (2), 78.856 (2), 68.043 (2)
V (Å^3)	2471.10 (11)
Z	2
Radiation type	Cu *K*α
μ (mm^{-1})	0.51
Crystal size (mm)	0.24 × 0.16 × 0.10
Data collection	
Diffractometer	Rigaku XtaLAB Synergy-Custom
Absorption correction	Multi-scan<br>*CrysAlis PRO* 1.171.41.110a (Rigaku Oxford Diffraction, 2021) Empirical absorption correction using spherical harmonics, implemented in SCALE3 ABSPACK scaling algorithm.
T_{min}, T_{max}	0.789, 1.000
No. of measured, independent and observed [$I > 2\sigma(I)$] reflections	29336, 9617, 8981
R_{int}	0.017
(sin θ/λ)$_{max}$ (Å^{-1})	0.626
Refinement	
$R[F^2 > 2\sigma(F^2)]$, $wR(F^2)$, S	0.045, 0.130, 1.03
No. of reflections	9617
No. of parameters	722
No. of restraints	126
H-atom treatment	H-atom parameters constrained
$\Delta\rho_{max}$, $\Delta\rho_{min}$ (e Å^{-3})	0.33, −0.40

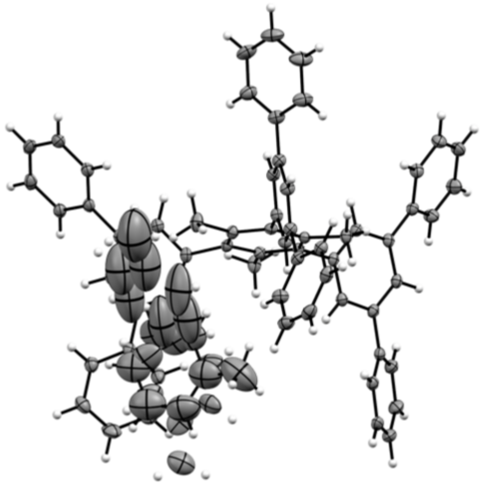

Supplementary Figure 20. An ORTEP diagram of **Ph^{VDW}• C_7H_8** with a probability level of 50 %.

Supplementary Table 13. Crystal structure information of **Ph^{VDW}•CH_2Cl_2.**

Crystal data	
Chemical formula	$C_{63}H_{48}\cdot 1(CH_2Cl_2)$
M_r	889.94
Crystal system, space group	Triclinic, $P\overline{1}$
Temperature (K)	100
a, b, c (Å)	11.2178 (3), 14.6142 (4), 17.3807 (3)
α, β, γ (°)	69.653 (2), 78.471 (2), 67.667 (2)
V (Å^3)	2463.65 (11)
Z	2
Radiation type	Cu $K\alpha$
μ (mm^{-1})	1.48
Crystal size (mm)	0.22 × 0.05 × 0.03
Data collection	
Diffractometer	Rigaku XtaLAB Synergy-Custom
Absorption correction	Multi-scan<br>*CrysAlis PRO* 1.171.41.110a (Rigaku Oxford Diffraction, 2021) Empirical absorption correction using spherical harmonics, implemented in SCALE3 ABSPACK scaling algorithm.
T_{min}, T_{max}	0.738, 1.000
No. of measured, independent and observed [$I > 2\sigma(I)$] reflections	28682, 9768, 8526
R_{int}	0.035
$(\sin \theta/\lambda)_{max}$ (Å^{-1})	0.626
Refinement	
$R[F^2 > 2\sigma(F^2)]$, $wR(F^2)$, S	0.056, 0.150, 1.04
No. of reflections	9768
No. of parameters	653
No. of restraints	81
H-atom treatment	H-atom parameters constrained
$\Delta\rho_{max}$, $\Delta\rho_{min}$ (e Å^{-3})	1.01, −0.50

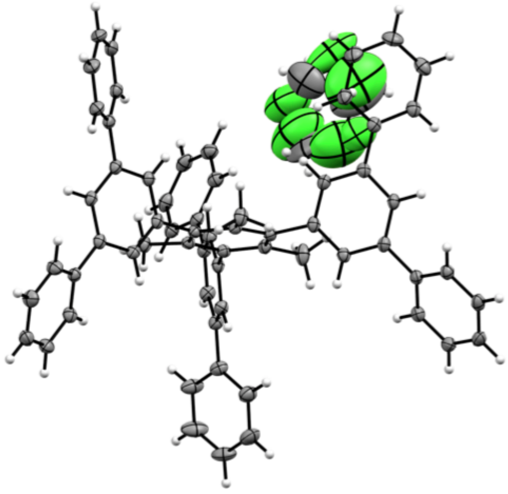

Supplementary Figure 21. An ORTEP diagram of **Ph^{VDW}• CH_2Cl_2** with a probability level of 50 %.

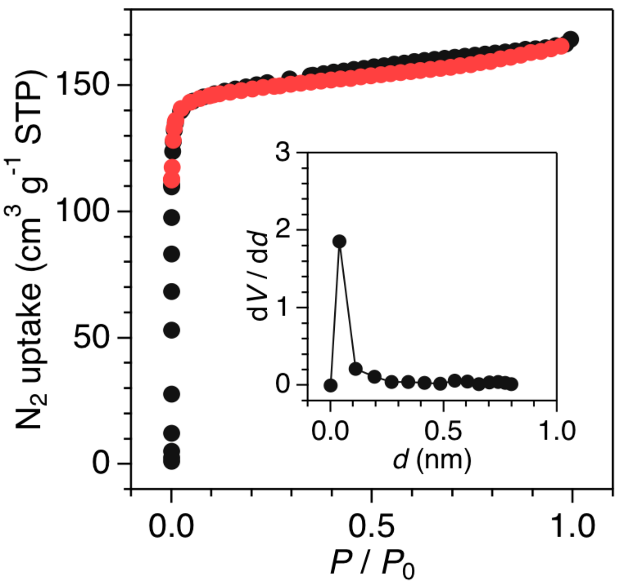

Supplementary Figure 22. N_2 adsorption (black circles) and desorption (red circles) isotherms of **Py^{open}·EtOAc** measured at 77 K. The BET surface area of **Py^{open}·EtOAc** calculated based on the isotherm is 597 cm^2 mol^{-1}. Inset represents the pore size distribution of **Py^{open}·EtOAc** calculated by means of micropore analysis (MP) method.

Supplementary Table 14. Relative permittivity (ε) and Hansen parameters[1] of the solvents utilized for the crystallization of **Py$_6$Mes**. The ε and Hansen parameters of the components of **Py$_6$Mes** are also listed for reference. [a] The ε values are obtained from a previous book.[2] [b] The ε values are obtained from a previous textbook.[3] [c] The ε values are obtained from a previous article.[4]

Entry	Crystallization Solvent	Permittivity	δ_D ($MPa^{0.5}$)	δ_P ($MPa^{0.5}$)	δ_H ($MPa^{0.5}$)	
1	MeOH	32.6[a]	15.1	12.3	22.3	Poorly soluble
2	EtOH	24.3[a]	15.8	8.8	19.4	
3	MeCN	37.5[a]	15.3	18.0	6.1	Porous Polymorphs
4	Butyronitrile	20.3[a]	15.3	12.4	5.1	
5	EtOAc	6.02[a]	15.8	5.3	7.2	
6	*i*PA	18.3[a]	15.8	6.1	16.4	
7	Acetone	20.7[a]	15.5	10.4	7.0	
8	1-PrCl	7.7[a]	16.0	7.8	2.0	
9	1-BuOH	15.8[a]	16.0	5.7	15.8	
10	THF	7.52[b]	16.8	5.7	8.0	
11	$CHCl_3$	4.81[a]	16.8	5.7	8.0	
12	Toluene	2.38[a]	18.0	1.4	2.0	
13	CH_2Cl_2	9.08[a]	18.2	6.3	6.1	
14	DMSO	47.2[b]	18.4	16.4	10.2	
15	γ-Butyrolactone	42.82[c]	19.0	16.6	7.4	
16	Mesitylene	2.27[a]	18.0	0.0	0.6	**Py$_6$Mes**
17	Benzene	2.27[a]	18.4	0.0	2.0	
18	Pyridine	12.3[a]	19.0	8.8	5.9	

Supplementary Table 15. Summary of PIEDA for **Py_6Mes** in **Py^{open}•MeCN**.

Crystallographic Axis	E^{ES} (kcal/mol)	E^{CT+mix} (kcal/mol)	E^{vdW} (kcal/mol)	E^{EX} (kcal/mol)	E^{att} (kcal/mol)	E^{total} (kcal/mol)
a	–6.92	–4.49	–21.24	10.68	–32.66	–21.97
b	–13.66	–12.09	–47.73	20.97	–73.48	–52.52
c	–11.58	–3.88	–13.14	8.78	–28.60	–19.82
total	–32.16	–20.46	–82.11	40.43	–134.73	–94.31

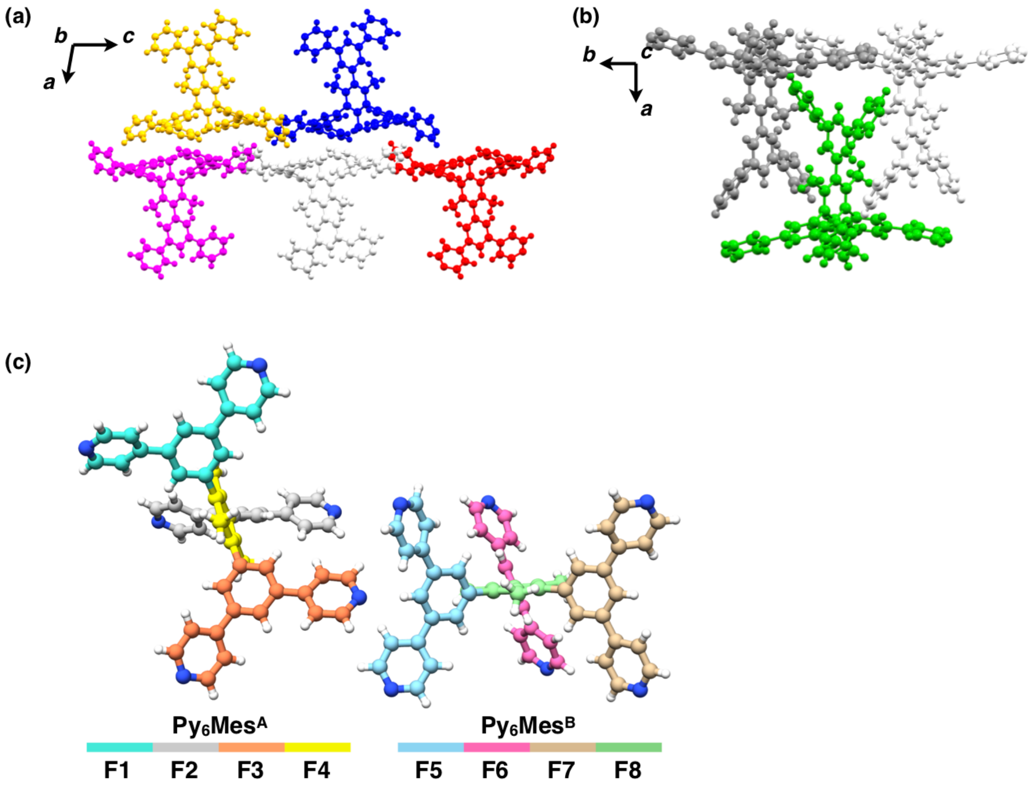

Supplementary Figure 23. (a) Symmetrically inequivalent contact pairs of **Py_6Mes** along the crystallographic *a* (molecules colored in red and pink) and *c* (molecules colored in yellow and blue) axes when focusing on a single molecule of **Py_6Mes** (colored in white). (b) Symmetrically inequivalent contact pairs of **Py_6Mes** along the crystallographic *b* (molecules colored in gray and green) axes when focusing on a single molecule of **Py_6Mes** (colored in white). (c) Fragmentation

of a contact pair of **Py$_6$Mes** in **Py^{open}•MeCN** (**Py$_6$Mes^{A}** and **Py$_6$Mes^{B}**) utilized for the calculation at FMO/RI-MP2 with 6-31+G(d) basis set. Each fragment is stained according to the color strips below.

Supplementary Table 16. Summary of calculated total system energies of **Py^{open}•MeCN** in a series of organic solvents with different relative permittivity ε.

Solvent	Vacuum	Toluene	$CHCl_3$	$C_2H_4Cl_2$	Acetone	MeOH
ε	1	2.38	4.81	10.37	20.56	32.66
Total system energy (kcal/mol)	–94.303	–101.54	–101.723	–101.904	–102.007	–102.047

3. Supporting references

(1) Hansen C. M. in *Hansen Solubility Parameters: A User's Handbook,* Second Edition (CRC Press, 2007).

(2) Maryott, A.A. & Smith E.R. in *Table of Dielectric Constants of Pure Liquids* (U. S. Government Printing Office, 1951).

(3) Bradley, J.-C. *et al.* in *Open Notebook Science Challenge: Solubilities of Organic Compounds in Organic Solvents* (Nature Precedings, 2010).

(4) Fornefeld-Schwarz, U. M. & Svejda, P. Refractive indices and relative permittivities of liquid mixtures of gamma-butyrolactone, gamma-valerolactone, delta-valerolactone, or epsilon-caprolactone plus benzene, plus toluene, or plus ethylbenzene at 293.15 K and 313.15 K and atmospheric pressure. *J. Chem. Eng. Data* **1999,** *44*, 597–604.
